# Oral Mucosal Lesions in Patients Attending Dermatology Outpatient Department of a Tertiary Care Center: A Descriptive Cross-sectional Study

**DOI:** 10.31729/jnma.8618

**Published:** 2024-06-30

**Authors:** Alina Karki, Varsha Manandhar, Rupak Maharjan, Alisha Maharjan

**Affiliations:** 1 Department of Dermatology, Nepal Armed Police Force Hospital, Balambu, Kathmandu, Nepal; 2 Department of Community Medicine, Manipal College of Medical Sciences, Fulbari, Pokhara, Nepal; 3 Department of ENT, Nepal Armed Police Force Hospital, Balambu, Kathmandu, Nepal

**Keywords:** *aphthous ulcer*, *dermatology*, *oral mucosal lesion*, *out-patient*

## Abstract

**Introduction:**

Oral mucosal lesions though mostly benign, may impair the quality of life of patients. Some may even progress to malignancies. Many physicians, including dermatologists, tend to skip oral examinations, missing many important diagnoses. Understanding the frequency and types of oral mucosal lesions in dermatological settings can help in early diagnosis, referral and adequate treatment. This study was done to determine demographic characteristics and clinical presentations of patients with oral mucosal lesions presenting to the out-patient department (OPD) of dermatology in Nepal Armed Police Force (APF) Hospital, Kathmandu.

**Methods:**

This cross-sectional descriptive study was conducted after obtaining the ethical approval from the Institutional Review Committee of Nepal APF Hospital. Retrospective data of 264 patients presenting with oral mucosal lesions to the dermatology OPD were collected from 1st January 2021 to 31st December 2023 by using a pre-formed proforma. Data was entered in SPSS software and descriptive statistics were computed.

**Results:**

Out of 13,832 cases, oral mucosal lesion was seen in 264 (1.90%) cases among which 153 (57.96%) cases were males with male female ratio of 1.37:1. Most common age group affected was 31-45 years 96 (36.36%). Buccal mucosa 86 (32.57%) was the commonest site involved followed by tongue 73 (27.65%). Aphthous ulcer 82 (31.06%) was the commonest lesion found followed by oral candidiasis 25 (9.46%) and oral lichen planus 24 (9.09%).

**Conclusions:**

Aphthous ulcer was the commonest oral mucosal lesion seen in patients visiting dermatology outpatient department of Nepal APF Hospital, with buccal mucosa being the commonest site affected.

## INTRODUCTION

Oral mucosal lesions (OML) include a wide range of conditions affecting the oral mucous membrane. They may have various presentations and may be traumatic, infective, inflammatory or neoplastic in origin.^1^ Most of these lesions may be benign, requiring only symptomatic treatment, but still may impair overall quality of life.^2^

Oral mucosa may show mainly the primary or sometimes, the only sign of various systemic diseases. Dermatological examination is considered incomplete without examination of the oral mucosa.^1,3^

The studies regarding prevalence of OML are very limited in patients visiting dermatology departments. These studies can contribute to raising awareness and provide valuable insights into the need for interdisciplinary collaboration between various oral health specialists facilitating improved patient care through early referral and appropriate management.

This study aimed to study the prevalence of oral mucosal lesions in patients visiting dermatology OPD of Nepal Armed Police Force (APF) Hospital and their clinical presentations.

## METHODS

This descriptive, cross-sectional study was conducted after getting ethical approval from the Institutional Review Committee of Nepal APF Hospital (IRC/NAPFH-002/2024). Purposive sampling technique was used to collect data of patients, irrespective of age and gender, who had visited the dermatology OPD of Nepal APF Hospital in Kathmandu from 1st January 2022 to 31st December 2023, with signs and symptoms suggestive of oral mucosal lesions. These cases were diagnosed by the duty dermatologists. Data was taken by retrospective manner from OPD registers of dermatology department which contained the particulars of the patients along with the clinical diagnosis provided by the dermatologists. Only the details of patients listed under the heading of 'oral lesions' were collected using preformed proforma, which was then cross-checked by the principal investigator. Patients with staining of teeth and mucosa were excluded from the study as it may be physiological or due to lifestyle in our settings.

The collected data was entered in Statistical Package for Social Sciences (SPSS) software for data analysis. Frequencies and proportions were calculated for categorical variables, while mean and standard deviation were calculated for continuous data.

## RESULTS

**Figure 1:**
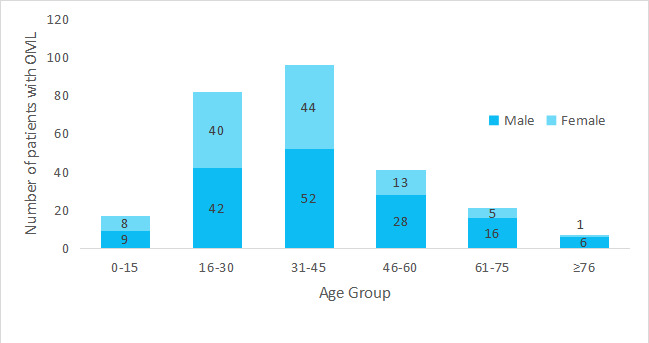
Age and sex-wise distribution of the patients with oral mucosal lesions (n = 264).

A total of 13,832 cases visited dermatology OPD during the data collection period, i.e. from 1^st^ January 2022 to 31^st^ December 2023. Out of these cases, 7476 (54.04%) were male and 6356 (45.96%) were females. Two hundred and sixty-four (1.90%) cases among these total cases were found to have oral mucosal lesions. Among those with OMLs, 153 (57.96%) were males and 111 (42.04%) were females, with a male-female ratio of 1.37:1. The participants ranged from 6 months to 89 years with mean age of 36.70 ± 17.05 years. Majority of patients 96 (36.36%) belonged to 3145 years group (Figure 1).

The most common oral mucosal lesion was aphthous ulcer, seen in 82 patients (31.06%), followed by oral candidiasis in 25 patients (9.46%) and oral lichen planus in 24 patients (9.09%) (Table 1).

**Table 1:** Distribution of individual oral mucosal lesions in the study participants (n = 264).

Oral lesions	Male (%)	Female (%)	Total (%)
Aphthous ulcer	33 (21.56)	49 (44.14)	82 (31.06)
Oral candidiasis	14 (9.15)	11 (9.90)	25 (9.46)
Oral lichen planus	17 (11.11)	7 (6.30)	24 (9.09)
Herpes Simplex infection	14 (9.15)	8 (7.20)	22 (8.33)
Geographical tongue	10 (6.53)	4 (3.60)	14 (5.30)
Fissure tongue	9 (5.88)	3 (2.70)	12 (4.54)
Glossitis	7 (4.57)	5 (4.50)	12 (4.54)
Vitiligo	7 (4.57)	5 (4.50)	12 (4.54)
Angular cheilitis	4 (2.61)	4 (3.60)	8 (3.03)
Pyogenic granuloma	6 (3.92)	2 (1.80)	8 (3.03)
Traumatic ulcer	5 (3.26)	3 (2.70)	8 (3.03)
Fixed Drug Eruption	4(2.61)	2 (1.80)	6 (2.27)
Leukoplakia	4(2.61)	2 (1.80)	6 (2.27)
Mucocele	4(2.61)	2 (1.80)	6 (2.27)
Oral submucosal fibrosis	6 (3.92)	-	6 (2.27)
Actinic cheilitis	1 (0.65)	3 (2.70)	4 (1.51)
Hemangioma	2 (1.30)	1 (0.90)	3 (1.13)
Behcet's disease	2 (1.30)	-	2 (0.75)
Pemphigus vulgaris	2 (1.30)	-	2 (0.75)
Squamous cell carcinoma	2 (1.30)	-	2 (0.75)
Total	153 (100.00)	111 (100.00)	264 (100.00)

In our study, buccal mucosa was the most commonly involved site, with its involvement seen in 86 (32.57%) patients, followed by tongue involvement in 73 (27.65%) patients (Table 2).

**Table 2:** Distribution of the OML patients according to the sites involved (n = 264).

Sites in oral cavity	n (%)
Buccal mucosa	86 (32.57)
Tongue	73 (27.65)
Labial mucosa	71 (26.89)
Palate	23 (8.71)
Others (gum, tonsillar area, etc.)	11 (4.16)

Among the patients with oral mucosal lesions, 98 (37.12%) had some kind of addiction out of which 81 (82.65%) were males and 17 (17.34%) were females. Among those with addiction, tobacco chewing 32 (32.65%) was the most common addiction followed by taking gutkha 20 (20.40%) smoking 18 (18.36%), betel nut chewing 14 (14.28%) and alcohol consumption 14 (14.28%).

Among 264 cases, 47 (17.80%) cases had some related or unrelated cutaneous manifestations along with oral manifestation. Among them 36 (76.59%) cases had specific skin conditions which presents with both oral and cutaneous lesions, and 11 (23.40%) had miscellaneous cutaneous manifestations unrelated to oral mucosal lesions. Lichen planus was the most common skin specific conditions affecting 10 (21.27%) cases, followed by vitiligo in 8 (17.02%), systemic lupus erythematosus in 6 (12.76%), psoriasis in 4 (8.51%), fixed drug eruptions in 4 (8.51%), pemphigus vulgaris in 2 (4.25%) and Behcet's disease in 2 (4.25%) cases.

## DISCUSSION

Out of 13,832 cases from 1^st^ January 2022 to 31^st^ December 2023, 264 (1.90%) cases were found to have oral mucosal lesions. Studies discussing prevalence of oral mucosal lesions are limited in Nepal, but this prevalence is similar to the studies conducted in different parts of India.^3-5^ Our study shows male predominance among the OML cases with male-female ratio of 1.37:1. This is similar to various studies conducted in Nepal and India.^2,6-12^ Male members of the community are more involved in habits of tobacco and betel nut chewing, smoking, which are the risk factors for OMLs,^13,14^ while due to social and cultural stigma for women in buying and using tobacco products, less number of females are usually involved. This could explain more number of male patients having OML. However, studies done by Mandadi et. al.^3^ Venereology and Leprosy (DVL and Arvind Babu et. al.^5^ NY in South India and Yao et. al.^15^ limited data are available for rural areas in China. We aimed to estimate the spectrum and frequency of OMLs and to identify their associated socioeconomic status (SES in China show that prevalence is more in female population. The health-seeking behaviors of the people in different regions could also affect the gender difference in the prevalence, as these are hospital-based studies.

In our study, most of the patients of OML are between the ages of 31 and 45 years, with the mean age of 36.70 years. This is similar to different studies which show that patients of OML usually belong to the third and fourth decades of life.^3,4,6,7,12,16,17^ Venereology and Leprosy (DVL This could be due to more involvement in risky habits of using betel nuts and tobacco products during this age group.

The most common sites involved in OML according to our study is buccal mucosa 86 (32.57%), followed by tongue 73 (27.65%) and labial mucosa 71 (26.89%). In majority of the studies done in Nepal^2,6,7^ and India^4,9,10,18^, similar results are seen with buccal mucosa being the most affected site.

Twenty different types of OMLs were detected in our study. Among the individual OMLs, aphthous ulcer 82 (31.06%) was the commonest, followed by oral candidiasis 25 (9.46%) and oral lichen planus 24 (9.09%). The most common lesions seem to vary according to different studies and different regions. A study by Goyal et. al. found the commonest lesion to be aphthous ulcer (44.50%) which is similar to our study.^18^ A study by Poudel et. al. found the commonest lesion to be mucocele (13.10%), followed by Squamous Cell Carcinoma (12.70%).^6^ However, in our study only 2 (0.75%) cases of Squamous Cell Carcinoma were present. This prevalence of SCC is much lower compared to 13.50% by Bajracharya et. al. and 9.50% by Acharya et. al. This difference could be because these studies were based on histopathological findings, while we only considered clinical presentations.^7,8^

In our study, 98 (37.12%) cases were found to have addictions which are considered risky behaviors for OML, with tobacco chewing present in 32 (32.60%) cases, followed by gutkha consumption in 20 (20.40%) and smoking in 18 (18.36%) patients. This result was very similar to that done in India by Jain et. al. where 32.25% of the patients were involved in tobacco chewing.^11^ In our study males were more involved in addictions than females this could also be the reason of higher cases of OML observed in males.

In our study, 47 (17.8%) cases of OML showed both cutaneous manifestations along with the oral mucosal manifestations. Among them specific skin conditions which presents with both oral and cutaneous lesions were seen in 36 (76.59%) of cases and 11 (23.40%) had miscellaneous cutaneous manifestations unrelated to oral mucosal lesions. Studies done by Asia et. al. showed OML cases with cutaneous manifestations in 37% of cases and Mandadi et. al. Venereology and Leprosy (DVL in 82% cases.^3,16^ This difference could be because both of these studies were from India and prevalence of various skin conditions may vary according to geographic area, climate and lifestyle of people. Moreover, there is paucity of research done from dermatology departments in Nepal. In our study, lichen planus 10 (21.27%) was the commonest specific skin conditions followed by vitiligo 8 (17.02%). Most of the studies show lichen planus, vitiligo, systemic lupus erythematosus (SLE), Steven Johnson's Syndrome (SJS), Discoid Lupus erytematosus (DLE), psoriasis as the major cutaneous manifestations along with OML. ^3-5,11,16,18^

There were a few limitations in this study. Only OML cases of dermatology department was considered, thus OML cases visiting dental or ENT department could have been missed. The findings of this study may not be generalizable as it is a single-centered study. Also, the cases were not diagnosed by single dermatologist, which could lead to variations in diagnosis.

## CONCLUSIONS

In our study, oral mucosal lesions were found to be present predominantly in male patients. Most of the patients were in the age group 31 to 45 years. Buccal mucosa was the most commonly affected site, with aphthous ulcer found to be the most common individual oral mucosal lesion.
